# Durability Degradation Mechanisms and Coupled Deterioration Behaviors of Geopolymer Concrete Under Multi-Hazard Service Environments: A Critical Review

**DOI:** 10.3390/ma19194050

**Published:** 2026-09-22

**Authors:** Jiale Li, Fei Yang, Ran Hai, Wenhao Mi, Cun Hui

**Affiliations:** 1 School of Intelligent Construction and Civil Engineering, Zhongyuan University of Technology, Zhengzhou 450007, Chinafeiyang@zut.edu.cn (F.Y.);; 2 School of Materials Science and Engineering, Henan University of Science and Technology, Luoyang 471023, China

**Keywords:** geopolymer concrete, alkali-activated materials, durability degradation mechanisms, coupled deterioration behaviors, multi-hazard service environments

## Abstract

Geopolymer concrete (GPC), here defined primarily as concrete based on low-calcium alkali-activated aluminosilicate binders, exhibits durability responses that depend strongly on precursor chemistry, gel assemblage, pore structure and exposure conditions; high-calcium or slag-rich systems are therefore discussed separately as related alkali-activated material (AAM) systems. This critical review synthesizes representative peer-reviewed evidence on freeze–thaw cycling, drying and autogenous shrinkage, elevated-temperature/fire exposure, chloride, sulfate and acid attack, carbonation, reinforcement corrosion and coupled multi-hazard deterioration. The evidence indicates that freeze–thaw damage is governed by pore-water phase change, cracking and transport-pathway formation, while chemical attack involves ion ingress, dealkalization, dealumination/decalcification and elemental leaching. Direct coupled evidence is strongest for freeze–thaw combined with chloride/salt or sulfate exposure; shrinkage–transport interactions are mainly correlation-supported, whereas carbonation–chloride–freeze–thaw-related corrosion remains largely a mechanism-based inference. Future work should prioritize standardized sequential multi-hazard protocols, chemistry-specific parameter databases and field-calibrated transport/corrosion models to support reliable service-life prediction and engineering design.

## 1. Introduction

Ordinary Portland cement (OPC) production is characterized by high energy consumption and considerable CO_2_ emissions. Against this background, research on low-carbon cementitious binders has gradually shifted from exploring their feasibility as cement substitutes to evaluating the reliability of their long-term service performance. In the strict terminology adopted in this review, geopolymer concrete (GPC) refers to concrete-scale composites prepared from predominantly low-calcium aluminosilicate precursors, such as fly ash or metakaolin, through alkaline activation. Slag-rich or calcium-rich alkali-activated concretes are discussed as related alkali-activated material (AAM) systems because their binding phases are commonly dominated by C-A-S-H or C-(N)-A-S-H gels rather than purely N-A-S-H-type geopolymer gels. Accordingly, GPC and related AAM systems exhibit promising advantages in cutting clinker consumption, promoting the valorization of industrial solid wastes, and alleviating adverse environmental impacts. Existing studies have shown that the engineering value of GPC is not limited to carbon reduction, but also depends on precursor reactivity, alkali-activation processes, gel microstructure, environmental performance and long-term service reliability [1,2,3]. Therefore, the practical application of GPC requires a durability-oriented assessment framework rather than evaluation based only on its low-carbon potential.

The durability performance of GPC and related AAM systems exhibits strong system-dependent characteristics. Recent reviews have emphasized that the durability of GPC cannot be evaluated only by mechanical strength, permeability or isolated exposure resistance. Instead, precursor composition, pore structure, exposure conditions, carbonation, freeze–thaw cycling, chemical attack and service-life-oriented indicators should be considered together [4,5,6]. Previous review studies have also revealed that precursor components, activator categories, curing regimes and pore-solution chemistry jointly govern the pore architecture, reaction products and transport behaviors of GPC and related AAM systems [7,8,9]. Accordingly, the durability of GPC cannot be generalized as intrinsically superior or inferior to that of OPC, and relevant discussions should be constrained by specific material systems, exposure environments and structural functional requirements. Within the present review, multi-hazard environments primarily denote a combination of service-induced deterioration conditions, encompassing freeze–thaw cycles, chloride-sulfate-acid attack, drying shrinkage, and carbonation, as well as their coupled interactions. Under practical in-service scenarios, temperature fluctuation, moisture variation, salt penetration, CO_2_ invasion, wet–dry cycles and mechanical loads frequently co-occur, giving rise to staged, path-dependent and amplified coupled damage. Freeze–thaw cycling modifies the phase state of pore water and impairs interfacial bonding integrity, which in turn generates transport channels facilitating the subsequent ingress of chloride ions, sulfate ions and CO_2_. Aggressive substances penetrating the matrix via interconnected pores and microcracks can trigger ion exchange and elemental leaching, as well as gel dealkalization, dealumination and decalcification. Drying shrinkage together with autogenous shrinkage is capable of inducing pre-existing microcracks prior to external attack, which reshapes material transport boundaries in advance. Carbonation reduces the alkalinity of pore solutions, alters gel phase assemblages and deteriorates the passivation condition for steel reinforcements. Consequently, the durability degradation of GPC under multi-hazard exposures should be interpreted as a sequential damage chain consisting of volume deformation, intensified mass transport, gel degradation, interfacial deterioration, reinforcement depassivation and performance degradation, rather than a straightforward superposition of isolated single-factor experimental observations. This manuscript is designed as a critical review rather than a PRISMA-based systematic review, and the literature discussed herein was selected from representative peer-reviewed studies related to GPC/AAM durability degradation, coupled deterioration mechanisms and service-life prediction. To avoid terminology ambiguity, alkali-activated materials (AAMs) are used as a broad category covering binders, pastes, mortars and concretes produced by alkaline activation of aluminosilicate or calcium–aluminosilicate precursors. The term “alkali-activated cement” refers more specifically to the binder system formed through alkaline activation, whereas alkali-activated concrete refers to concrete-scale composites containing aggregates. Geopolymers are treated as a subset of low-calcium alkali-activated binders mainly dominated by N-A-S-H-type gels, while high-calcium or slag-rich alkali-activated systems generally contain C-A-S-H or C-(N)-A-S-H gels. Accordingly, although the title focuses on geopolymer concrete, this review also incorporates evidence from broader AAM paste, mortar and concrete systems only when such evidence provides mechanistic support for interpreting GPC durability. Evidence from these broader systems is used with caution, and its extrapolation to concrete-scale GPC is discussed by considering material scale, precursor chemistry, aggregate restraint and interfacial transition zones.

Building upon the above-mentioned research foundation, the present review focuses on the durability-related degradation mechanisms of GPC under multi-hazard environments, while incorporating relevant AAM evidence only where it supports mechanistic interpretation. Firstly, the distinctions in damage mechanisms between GPC and OPC are analyzed from the standpoints of gel assemblages, pore architecture and pore-solution chemistry. Secondly, the damage evolution behaviors of GPC under individual actions of freeze–thaw cycling, drying shrinkage, chemical attack and carbonation are elaborated. Thirdly, this work summarizes the available evidence and inferred mechanisms associated with coupled deterioration, including freeze–thaw–chemical attack, shrinkage–transport interaction, and carbonation–chloride-related reinforcement corrosion risk. Ultimately, a multi-indicator evaluation framework oriented toward service-life prediction is put forward. This review intends to elucidate the degradation pathways governing GPC durability under multi-hazard exposures, which are jointly controlled by material composition, pore microstructure, crack propagation and the transport of aggressive agents. It further seeks to deliver technical references for the engineering implementation of GPC in cold-climate zones, salt-erosion-prone regions and reinforced concrete structural systems. To clarify the novelty of this review, representative reviews cited in this manuscript are compared in Table 1. The comparison highlights their material scope, main focus, coverage of coupled deterioration, and the remaining gaps addressed by the present review.

## 2. Damage Characteristics of GPC and Related AAM Systems

### 2.1. Gel Products and Pore-Structure Features

The durability of GPC and related AAM systems is governed by the coupled effects of precursor chemistry, gel assemblage, pore architecture, pore-solution chemistry and aggregate/interface characteristics. As summarized in Figure 1, environmental actions such as freeze–thaw cycling, chloride/sulfate/acid attack, drying shrinkage, carbonation and elevated-temperature exposure interact with internal material features, including gel type, pore structure, pore solution and aggregate/ITZ effects. These interactions may trigger pore-structure change, microcracking, transport-pathway evolution, gel degradation, reinforcement depassivation/corrosion risk and mechanical deterioration. Therefore, the durability response of GPC/AAM systems should be interpreted using a multi-indicator framework rather than a single macroscopic index.

Low-calcium GPC systems derived mainly from fly ash or metakaolin are generally characterized by N-A-S-H-type aluminosilicate gels, whose stability is related to the connectivity of SiO_4_ and AlO_4_ tetrahedra and the charge-balancing role of alkali-metal cations such as Na^+^ and K^+^. Because of their limited soluble calcium content, these systems may exhibit favorable resistance under certain acid or sulfate exposures. However, their long-term alkalinity retention and steel-protection capacity remain uncertain, especially when carbonation, chloride ingress or cracking is involved. In contrast, high-calcium or slag-rich AAM systems tend to form C-A-S-H or C-(N)-A-S-H gels. These gels can promote early reaction and matrix densification, but they may also increase sensitivity to autogenous shrinkage, decalcification, carbonation and pore-structure reconstruction. Blended GPC/AAM systems, such as fly ash–slag mixtures, usually contain multiple gel phases, unreacted precursors and metastable reaction products; therefore, their durability depends strongly on Ca/Si/Al ratios, activator formulation, curing regime and alkali-activation degree.

Pore microstructure is a key intermediate linking alkali-activation reactions with macroscopic durability performance. However, its influence cannot be adequately represented by total porosity alone. Studies on slag/fly ash-based AAMs and low-calcium geopolymer systems indicate that pore structure is controlled by precursor reaction kinetics, gel development, residual particles and modifier dispersion [14,15,16,17]. Compared with total porosity, pore-size distribution, critical pore radius and pore connectivity provide more useful information for interpreting strength development, permeability, shrinkage behavior and long-term degradation. Microstructural observations reported by Bhagath et al. [18] further show that reaction gels, unreacted precursor particles, local pore defects and interfacial regions may coexist in low-calcium fly ash-based GPC. These heterogeneous regions can act as preferential sites for crack initiation and aggressive-agent transport, but their influence should be interpreted together with specimen scale, aggregate restraint and exposure conditions.

In summary, the durability advantages of GPC should not be simply attributed to low porosity or high mechanical strength. Long-term performance is controlled by the combined effects of gel stability, pore-size distribution, pore connectivity, pore-solution chemistry, aggregate/ITZ characteristics and environmental boundary conditions. Future studies should establish system-dependent quantitative relationships among gel composition, pore architecture, pore-solution chemistry and durability indicators, with particular attention to low-calcium GPC systems, high-calcium or slag-rich AAM systems and blended GPC/AAM systems.

### 2.2. Mechanistic Differences Between GPC and OPC

In OPC systems, primary hydration phases including Ca(OH)_2_, C-S-H gels and ettringite dominate matrix alkalinity maintenance, strength evolution and resistance against aggressive media. Acid attack principally triggers the dissolution of Ca(OH)_2_ and progressive decalcification of C-S-H gels, while sulfate attack generally induces the formation of expansive gypsum and ettringite, which initiates structural degradation. Accordingly, low-calcium GPC deteriorates mainly through alkali leaching, gel dealkalization, dealumination and progressive destabilization of the aluminosilicate network. In calcium-rich or slag-rich AAM systems, decalcification of C-A-S-H or C-(N)-A-S-H gels and the associated pore-structure rearrangement may additionally contribute to deterioration.

Evidence from numerical modeling, comparative durability tests and modifier-related studies indicates that the deterioration difference between GPC and OPC is closely associated with pore-water phase transition, ion transport, gel chemistry, pore-solution environment and interfacial transition zone characteristics [19,20,21]. These findings suggest that the durability comparison between GPC and OPC should be interpreted from a coupled microstructure–chemistry–transport perspective rather than from macroscopic strength alone. These findings further confirm that the performance disparities between GPC and OPC are not limited to macroscopic mechanical properties, but originate from essential differences in reaction product compositions, pore-solution chemical environments and internal mass transport mechanisms.

Such fundamental microstructural differences endow GPC with distinct deterioration pathways compared with OPC under aggressive service environments. Low-calcium GPC systems feature limited soluble calcium contents, which mitigates the extensive dissolution of calcium-bearing phases during acid attack; however, the migration of alkali metal ions and insufficient alkalinity buffering capacity of pore solutions may undermine the long-term structural stability of the matrix. By contrast, high-calcium or slag-rich AAM systems with abundant C-A-S-H and C-(N)-A-S-H gels exhibit improved early-age mechanical performance and matrix compactness, yet these gel phases are vulnerable to decalcification and pore structure rearrangement under carbonation, acid attack and sulfate ingress. In terms of frost resistance, the freeze–thaw durability of OPC concrete is primarily controlled by air-void spacing, critical saturation degree and interfacial bonding status, whereas the freeze–thaw performance of GPC is further governed by gel-bound water states, pore-solution concentration, residual activator components and additive-matrix compatibility.

Accordingly, the generalized conclusion that GPC possesses superior durability to OPC is not universally applicable. The durability advantages of GPC are highly condition-dependent and are valid only under specific precursor compositions, calcium content levels, alkali-activation regimes, curing protocols, exposure media and structural service scenarios. Future research should establish systematic comparative evaluation matrices classified by gel system types and aggressive environment categories, rather than conducting simplistic and generalized durability comparisons between GPC and OPC concrete. To clarify the material basis of the reviewed studies and avoid treating different GPC/AAM systems as interchangeable, the representative material systems, calcium levels, binder or activator features, curing/exposure conditions and reported durability responses are summarized in Table 2.

### 2.3. Scale Effects of Aggregates and Reinforcement in GPC/AAM Systems

In practical engineering, alkali-activated binders and geopolymer binders are rarely used as isolated paste systems. They are normally combined with fine aggregates, coarse aggregates and, in reinforced members, steel reinforcement. Therefore, durability mechanisms identified at the binder, paste or mortar scale should not be directly generalized to concrete-scale GPC/AAM systems without considering aggregate-related restraint, interfacial transition zones and structural boundary conditions. Recent studies on MK–GGBFS geopolymer mortar–aggregate interfaces indicate that the interfacial transition zone can influence moisture diffusion, chloride transport and impermeability, suggesting that aggregate type, aggregate geometry, ITZ thickness and local pore structure should be incorporated into concrete-scale durability interpretation [78].

In reinforced members, the binder matrix, aggregates and reinforcement act as an integrated load-bearing system rather than independent phases. Bond behavior between reinforcement and GPC directly affects load transfer, crack resistance and structural integrity; therefore, bond strength, bond–slip response, development length, cover depth, bar diameter, confinement condition and ITZ characteristics should be considered when translating material-scale durability results into reinforced-member performance [79]. Recent studies on reinforced GPC columns further show that structural performance is governed by material composition, reinforcement configuration, concrete strength, load eccentricity, confinement effect, loading condition and environmental exposure. Accordingly, future durability studies should move from isolated binder degradation toward multi-scale assessment linking binder chemistry, aggregate-controlled transport, ITZ cracking, steel–matrix bonding, reinforcement corrosion and residual load-bearing capacity [80].

### 2.4. Durability Evaluation Indicators

A comprehensive durability evaluation of GPC under multi-hazard conditions requires a paradigm shift from single macroscopic indices to a hierarchical multi-level indicator system. At the macroscopic performance level, mass loss ratio, residual compressive strength and relative dynamic elastic modulus characterize surface scaling behavior, degradation of load-bearing capacity and variations in internal structural integrity, respectively. At the mass transport level, key parameters including chloride diffusion coefficient, water absorption rate, gas diffusion coefficient and electric charge permeability quantify the ingress and migration capability of water, aggressive ions and gaseous agents within the matrix. At the volume stability level, drying shrinkage, autogenous shrinkage, shrinkage-induced stress and crack width describe the pre-existent deformation and initial defect boundaries generated prior to external environmental attack. At the microchemical and reinforcement protection level, critical indicators cover pore-solution pH value, soluble alkali content, gel microstructure evolution, and elemental leaching behavior, as well as electrochemical parameters of steel reinforcement, including open-circuit potential, corrosion current density and polarization resistance.

These multi-scale indicators do not function as independent parallel descriptors; instead, they correspond to sequential stages throughout the entire deterioration chain. Mass loss effectively reflects surface damage but exhibits low sensitivity to early-stage internal microcracking. Strength retention intuitively evaluates the degradation of mechanical performance yet fails to reveal the formation and evolution of internal permeable pathways. Although electromigration tests enable rapid characterization of ion transport behavior, their applicability and interpretation for alkali-activated materials are substantially influenced by pore-solution conductivity, ion binding effects and specimen pretreatment procedures. Accordingly, accurate and reliable durability evaluation of GPC necessitates the integrated consideration of macroscopic mechanical performance, mass transport characteristics, volume stability, microchemical structural evolution and reinforcement protection capability. Such a multidimensional evaluation framework can further provide fundamental support for refined service-life prediction of low-carbon geopolymer concrete under complex multi-hazard service environments.

## 3. Damage Evolution Under Physical Exposure Conditions

### 3.1. Pore Coarsening and Crack Propagation Under Freeze–Thaw Cycling

Freeze–thaw cycling constitutes a dominant deterioration trigger for GPC deployed in cold-region engineering. Frost-induced damage is not an abrupt failure occurring upon the completion of freeze–thaw cycles; instead, it evolves via a progressive process encompassing pore-water migration, build-up of ice-crystal pressure, cracking within interfacial transition zones, crack interconnection, elevated mass-transport capacity and consequent mechanical performance decay. Under cyclic freeze–thaw loading, repeated phase transformation of pore water generates frost-heaving pressure and hydraulic pressure, which preferentially impose stresses on zones featuring larger pore dimensions, high pore connectivity or inferior interfacial bonding. With accumulating freeze–thaw cycles, intrinsic pores undergo gradual coarsening, interfacial defects propagate into microcracks, and interconnected permeable pathways are ultimately established within the matrix.

Zhang et al. [22] reported that metakaolin can improve the frost resistance of alkali-activated slag systems by tailoring reaction assemblages and refining pore-size distribution. Together with studies on alkali-activated slag–coal gangue concrete and polypropylene-fiber-modified geopolymer mortar, the available evidence indicates that freeze–thaw cycling can progressively shift the pore network from a refined state toward a coarser and more connected structure, reduce matrix compactness, increase chloride-ingress susceptibility and promote microcrack propagation [22,81,82]. Fiber reinforcement may improve crack resistance under low-temperature exposure by restraining crack extension and enhancing matrix toughness. Microstructural observations reported by Pilehvar et al. [83] further suggest that interfacial transition zones, particle boundaries and localized pore defects can act as preferential initiation sites for frost-induced cracking. Therefore, freeze–thaw deterioration in GPC/AAM-related systems should be interpreted as a progressive damage process involving pore coarsening, interfacial cracking, crack connectivity and transport-pathway formation.

Macroscopic freeze–thaw tests on GPC-related systems further indicate that mass variation, stiffness attenuation, crack development and strength degradation may evolve concurrently with increasing cycle number [84,85]. These observations provide the macroscopic basis for the multi-indicator frost-damage assessment discussed in Section 3.2.

### 3.2. Mass Loss and Mechanical Performance Degradation Under Freeze–Thaw Cycling

Under freeze–thaw cycling, the macroscopic deterioration of GPC/AAM systems is commonly reflected by mass loss, strength degradation and reductions in relative dynamic elastic modulus. Mass loss rate intuitively reflects surface scaling and particle exfoliation; nevertheless, its variation generally lags behind the evolution of internal pore connectivity and microcrack propagation, making it inadequate as a standalone indicator for evaluating early-stage freeze–thaw damage. Studies on fly ash-based GPC, slag-rich AAMs and related geopolymer systems collectively indicate that freeze–thaw resistance is strongly controlled by mineral admixtures, mixture design parameters and curing regimes [25,26,27]. These factors influence mass stability and residual mechanical performance by regulating gel microstructure, pore-solution chemistry and internal pore connectivity. Among them, the SiO_2_/Al_2_O_3_ molar ratio is particularly important because it influences the formation and stability of aluminosilicate gels, the refinement of pore structure and the connectivity of transport pathways. An appropriate SiO_2_/Al_2_O_3_ molar ratio may reduce freeze–thaw-induced mass loss and stiffness degradation, suggesting that the Si/Al ratio should be regarded not only as a strength-related parameter, but also as a key mixture-design variable governing the frost durability of GPC/AAM systems.

Relative dynamic elastic modulus, ultrasonic pulse velocity, water absorption and chloride transport properties can compensate for the low sensitivity of mass loss to early internal microdamage. Evidence from reinforced GPC beams, cement-kiln-dust-based alkali-activated matrices and recycled aggregate alkali-activated concrete indicates that frost resistance is jointly affected by saturation degree, freeze–thaw regime, pore microstructure, material scale, aggregate type and transport characteristics [10,28,86,87,88]. These studies also suggest that different durability indicators may respond asynchronously under the same freeze–thaw condition: mass loss mainly reflects surface scaling or material detachment, RDEM and ultrasonic pulse velocity are more sensitive to internal cracking and stiffness degradation, while water absorption and chloride transport parameters characterize the evolution of connected pores and transport pathways. Therefore, freeze–thaw durability should be evaluated using a multi-indicator framework, and findings from different GPC/AAM-related systems should not be directly generalized without considering binder chemistry, aggregate condition and specimen scale.

For frost resistance enhancement, fibers and nanomaterials improve the freeze–thaw stability of GPC via crack bridging, pore refinement and interfacial reinforcement. Li et al. [89] adopted modified multi-walled carbon nanotubes and polyvinyl alcohol (PVA) fibers to synergistically strengthen fly ash-based geopolymers and characterized post-freeze–thaw microstructural morphology via field-emission scanning electron microscopy. Modified carbon nanotubes fill internal pores and localized defects, bridge microcracks, and form tight entanglement with geopolymer gels, thereby reinforcing interfacial bonding and local fracture resistance. By contrast, PVA fibers dissipate freeze–thaw-induced internal stress at the macroscopic scale and restrain crack extension. These results reveal that the frost-resistant modification mechanism of fibers and nanomaterials relies not merely on strength enhancement, but on multi-scale crack regulation through pore filling, crack bridging and interfacial interlocking, which mitigates water penetration and suppresses cumulative freeze–thaw damage.

Air-void microstructure serves as another essential design indicator for GPC frost performance. Air-entraining agents function not merely by increasing porosity, but by generating stable, evenly distributed and size-optimized microvoids within the matrix. These voids provide buffer spaces for volumetric expansion of frozen water and relieve salt crystallization pressure. Investigations on ambient-cured slag-fly ash alkali-activated materials, alkali aluminosilicate cement concrete and alkali-slag concrete have verified that bubble stability, void spacing, matrix compactness, mixture proportion and mixing water condition jointly govern frost resistance [23,90,91,92]. Such characteristics indicate that the frost optimization of GPC requires multi-objective coordination of air-void structure, matrix strength and pore connectivity, rather than single-parameter adjustment.

In terms of testing methodology, frost resistance of GPC/AAM systems should not be assessed only by standalone freeze–thaw results. Studies on alkali-activated concrete, high-alkali slag mortars and OPC air-entrained concrete suggest that frost durability depends on material compatibility, pore structure, strength degradation and stable air-void formation [24,29,30]. Winnefeld et al. [31] provided round-robin evidence showing that salt-frost scaling of alkali-activated concretes is highly sensitive to curing and specimen preconditioning. As shown in Figure 2, nominally comparable mixtures exhibited markedly different cumulative scaling responses among participating laboratories. These differences should therefore be interpreted primarily as evidence of protocol sensitivity rather than as a universal ranking of intrinsic frost resistance. Accordingly, curing history, pre-saturation conditions and other test boundary parameters should be explicitly reported before cross-study comparison. These findings indicate that GPC frost evaluation should avoid simple comparison of scaling mass at fixed cycle numbers. Specimen pretreatment, saturation regime, salt solution environment and test termination criteria should be fully documented. Synchronous monitoring of ion leaching, mass variation, strength retention and surface damage is required to comprehensively characterize macroscopic deterioration after freeze–thaw exposure [93,94].

Overall, frost-resistance results should be interpreted within clearly defined material and testing boundaries. Precursor and activator characteristics, saturation state, freeze–thaw protocol, salt environment, air-void structure and termination criteria should therefore be consistently reported to enable reliable cross-study comparison.

### 3.3. Drying Shrinkage and Shrinkage-Induced Cracking Mechanisms

Drying shrinkage represents a pre-induced microstructural defect mechanism that is frequently underestimated in GPC durability research because it can modify crack morphology and transport boundaries before subsequent environmental exposure. Studies on fly ash/slag-based GPC and alkali-activated composites indicate that drying shrinkage is governed by curing protocol, activator dosage, precursor proportion and gel-phase composition [32,33,95]. Its durability significance lies not only in dimensional variation, but also in restrained microcracking that may increase pore/crack connectivity and facilitate subsequent moisture, chloride, sulfate and CO_2_ transport.

Drying shrinkage and autogenous shrinkage can both initiate microcracking, but they differ in driving mechanisms, development stages and environmental dependence. Studies on alkali-activated fly ash–slag pastes and alkali-activated slag systems indicate that autogenous shrinkage is closely related to internal relative humidity reduction, capillary pressure, gel water redistribution and gel microstructure evolution [34,35,36,96,97]. In high-calcium or slag-rich AAM systems, internal self-desiccation and deformation or rearrangement of C-A-S-H-type gels may contribute to irreversible shrinkage and early-age cracking. Because much of this evidence is derived from paste- or mortar-scale systems, its extrapolation to blended GPC should account for aggregate restraint, interfacial transition zones and drying boundary conditions. Accordingly, autogenous shrinkage is mainly governed by internally driven moisture redistribution and early-age reactions, whereas drying shrinkage is more strongly controlled by external humidity exchange and moisture loss.

Shrinkage mitigation should therefore focus not only on reducing free deformation, but also on controlling internal humidity, crack development and transport pathways. Reported mitigation strategies include pore-surface regulation and internal curing using vegetable oil, pre-wetted lightweight aggregates, or superabsorbent polymers [98,99,100], as well as matrix modification using nano-SiO_2_, limestone powder, shrinkage-reducing admixtures, MgO-based expansive agents and nanocellulose [101,102,103,104,105,106]. Their effectiveness remains strongly dependent on dosage, absorption–desorption behavior, alkaline stability and compatibility with the GPC/AAM matrix. Therefore, shrinkage mitigation should be assessed together with strength development, crack restraint, pore connectivity and subsequent resistance to aggressive-agent transport.

### 3.4. Elevated Temperature and Fire-Induced Damage

Elevated temperature and fire exposure should be considered as an additional durability-related scenario for GPC/AAM systems. Unlike freeze–thaw cycling or chloride/carbonation exposure, thermal damage is mainly governed by rapid moisture migration, pore-pressure build-up, thermal incompatibility between aggregates and binder matrix, gel dehydration, phase transformation, thermal cracking and possible spalling. Existing reviews indicate that the high-temperature performance of GPC/AAM systems is strongly dependent on precursor chemistry, calcium content, aggregate type, activator formulation, curing regime, heating rate, peak temperature and cooling procedure [107,108,109]. Therefore, fire resistance should not be regarded as an inherent advantage of all GPC systems, but should be interpreted as a material system-dependent property.

For low-calcium fly ash-based GPC, moderate heating may promote further alkali-activation reactions or local matrix densification, which can partly contribute to residual strength retention. However, at higher temperatures, evaporation of physically bound water, gel dehydration, pore coarsening and thermal microcracking may gradually dominate the damage process. For high-calcium or slag-rich AAM systems, C-A-S-H or C-(N)-A-S-H type gels may undergo dehydration or structural rearrangement, making residual performance more sensitive to calcium content and thermal history. Sarker et al. [110] provided representative evidence that cracking, spalling and residual strength of fly ash-based geopolymer concrete are closely related to fire exposure conditions. Accordingly, post-fire durability should be evaluated using multiple indicators, including mass loss, residual strength, elastic modulus, crack morphology, spalling tendency, pore-structure evolution and phase assemblage. Future studies should establish standardized heating/cooling regimes and further examine the combined effects of fire exposure with pre-existing shrinkage cracks, chloride ingress, carbonation and reinforcement corrosion.

## 4. Damage Evolution Under Chemical Attack

### 4.1. Damage Mechanisms Under Chloride, Sulfate and Acid Attack

Under chemical-attack conditions, the degradation mechanisms of GPC/AAM systems vary with aggressive-agent categories and matrix calcium levels, given that chloride, sulfate, inorganic-acid and organic-acid exposures trigger distinct transport patterns, secondary products and elemental-leaching routes. Comparative studies on GPC, OPC, slag-rich AAM systems and geopolymer mortars indicate that chemical attack is governed by aggressive-agent type, calcium content, gel-network stability and elemental leaching behavior [111,112,113,114]. In contrast to OPC, whose deterioration is strongly associated with calcium-bearing phase dissolution and expansive products, GPC/AAM systems mainly degrade through alkali leaching, gel dealkalization, dealumination/decalcification and pore coarsening. Therefore, evidence from mortar- and binder-scale systems is used here primarily for mechanistic interpretation rather than direct quantitative prediction of concrete-scale GPC performance.

Soluble-calcium availability and gel-network integrity are two key factors controlling the different chemical-attack responses of GPC/AAM systems and OPC. Sata et al. [37] showed that mass variation and strength degradation do not always evolve synchronously under sulfate or sulfuric-acid exposure, indicating that mass change alone is insufficient for durability assessment. He et al. [115] provided mineralogical evidence from a slag-rich alkali-activated concrete containing NaCl, gypsum and quicklime. As shown in Figure 3, XRD analysis after sulfate exposure identified portlandite, Friedel’s salt, calcite, quartz, gypsum, ettringite, sodalite and dolomite. Friedel’s salt reflects chloride binding, whereas the coexistence of multiple calcium- and aluminosilicate-bearing phases demonstrates the complex reaction environment of this high-calcium system. Importantly, gypsum and ettringite were also associated with the original composite-activated matrix; therefore, their detection alone cannot be regarded as direct evidence of sulfate-induced precipitation. Phase identification should instead be interpreted together with control specimens, microstructural observations and macroscopic deterioration indicators.

Across acid resistance studies, the reported performance of GPC/AAM systems is strongly dependent on precursor chemistry, acid type, solution concentration, pore refinement and modification strategy [11,38,39,115]. Some blended-ash GPC systems can retain relatively high residual strength under sulfuric acid exposure, but acid-induced degradation generally proceeds through dealkalization, dealumination/decalcification, elemental leaching and pore coarsening. Organic and inorganic acids should not be treated as equivalent exposure conditions, because complexation reactions and ion-leaching routes may differ substantially. Pore-refining modifiers may also affect subsequent carbonation resistance by altering microstructure and transport pathways, as reported for nano-SiO_2_-modified geopolymer recycled concrete [40].

In summary, the chemical resistance of GPC/AAM systems is condition-dependent rather than intrinsic. Its performance is controlled by the combined effects of calcium availability, gel-network stability, pore connectivity, aggressive-agent type and exposure duration. Future studies should report these boundary conditions consistently and establish separate mechanism–indicator relationships for chloride, sulfate, inorganic-acid and organic-acid exposures.

### 4.2. Mass Change and Structural Deterioration Under Chemical Attack

Under chemical attack conditions, mass variation represents a comprehensive result of aggressive medium ingress, secondary product accumulation, surface dissolution and matrix particle exfoliation. Okoye et al. [41] stated that the durability performance of silica fume-modified fly ash-based GPC is highly dependent on pore microstructure evolution and reaction product characteristics. Relevant investigations on sulfate attack under coastal wetting-drying cycles, acid attack of lightweight alkali-activated structural concrete, and mild acid deterioration of waste glass powder-calcium aluminate geopolymer mortar consistently confirm that mass change originates from the superposition of reaction product filling, salt crystallization retention and matrix dissolution [42,43,44]. Accordingly, mass increment cannot be regarded as a reliable indicator of improved durability, and negligible mass fluctuation does not equate to stable internal microstructure. Individual mass variation curves may even cause misleading evaluation conclusions without supplementary evidence from elemental leaching analysis, pore structure characterization and strength retention monitoring.

Comprehensive durability assessment should integrate mass variation, residual strength, pore-structure evolution, phase transformation and elemental leaching rather than rely on a single response index. Studies on metakaolin-based geopolymer mortar, fly ash–slag blended GPC and fluidized-bed fly ash–metakaolin geopolymers indicate that chemical exposure can induce progressive degradation, while residual performance depends strongly on precursor composition, soluble calcium content, additive type, exposure chemistry and gel-network stability [45,46,47]. These findings suggest that mass change and residual strength are useful for evaluating overall deterioration, but they cannot fully reveal the underlying corrosion process. Therefore, microstructural and mineralogical evidence is required to identify gel degradation, secondary product formation, pore enlargement and transport-pathway evolution.

Yang et al. [116] provided representative SEM evidence for low-calcium Class F fly ash GPC activated with 8 M NaOH. As shown in Figure 4, the unexposed F-8 matrix contains unreacted fly ash particles, voids and local microstructural defects, whereas after 98 d of cyclic sulfuric-acid exposure the matrix becomes visibly looser and secondary gypsum is observed. These observations suggest that acid deterioration involves simultaneous degradation of the aluminosilicate matrix, secondary crystallization and progressive microstructural heterogeneity. Therefore, the presence of secondary products should not be interpreted solely as beneficial pore filling, because their effect depends on crystal morphology, distribution and the stability of the surrounding gel network.

### 4.3. Alkalinity Reduction and Gel-Structure Changes Under Carbonation

Carbonation critically affects the steel-protection performance of reinforced GPC/AAM systems by altering not only carbonation depth, but also pore-solution alkalinity, soluble alkali reserves, gel stability and transport characteristics. Available studies indicate that carbonation resistance is strongly dependent on precursor chemistry, slag or calcium content, curing regime and pore structure [12,48]. In low-calcium fly ash-based GPC and related AAM systems, CO_2_ ingress may induce alkali leaching, pH reduction and aluminosilicate gel destabilization, whereas high-calcium or slag-rich systems may additionally undergo gel decalcification and carbonate precipitation. Evidence from natural carbonation, accelerated carbonation and round-robin testing further shows that carbonation behavior cannot be directly generalized from OPC or compared across studies without considering pore-solution chemistry, precursor type and testing procedure [49,50,51,52]. Therefore, carbonation assessment should move beyond standalone carbonation-depth measurements and integrate alkalinity retention, gel-phase evolution, pore-structure changes and reinforcement-protection indicators.

For reinforced GPC/AAM systems, carbonation risk cannot be reliably evaluated solely by pH value or carbonation depth; instead, carbonation front evolution, pore-solution composition and steel electrochemical status require integrated assessment. Xu et al. [13] reviewed steel passivation and depassivation behaviors in AAM and clarified that pore-solution pH, sulfide content, chloride concentration and chemical composition collectively dominate reinforcement protection efficiency. Ma et al. [117] suggested that steel open-circuit potential and corrosion current density under carbonation exposure should be analyzed together with pore-solution properties. Parametric carbonation models, accelerated carbonation tests with varied activators, and carbonation investigations on slag-calcined clay systems further prove that precursor composition, pore structure and thermodynamic stability jointly determine carbonation product assemblages and carbonation front migration [118,119,120,121].

In conclusion, carbonation depth merely reflects the superficial degree of GPC carbonation deterioration. Low-calcium N-A-S-H systems primarily undergo alkali ion leaching, gel dealkalization and aluminosilicate network reconstruction during carbonation. By contrast, high-calcium C-A-S-H and C-(N)-A-S-H systems experience simultaneous gel decalcification, carbonate deposition and pore structure reorganization. Future studies should establish a unified evaluation framework for steel protection performance by integrating pore-solution pH, soluble alkali content, chloride binding state, gel microstructure and steel electrochemical responses. Based on the single-hazard deterioration processes discussed above, the dominant mechanisms, recommended durability indicators and critical boundary conditions for different exposure types are summarized in Table 3.

## 5. Durability Degradation Mechanisms Under Multi-Factor Coupled Actions

Before discussing specific coupled deterioration pathways, this review classifies the available evidence into three levels: Level I represents direct evidence from simultaneous or sequential multi-hazard exposure tests; Level II refers to correlation-supported evidence between durability-related properties; and Level III denotes mechanism-based inference from separate single-hazard studies. This classification is used to distinguish experimentally supported interactions from hypotheses requiring further validation and to avoid overstating coupled degradation mechanisms. The evidence level, exposure mode, interaction type, dominant mechanism and limitation of representative pathways are summarized in Table 4.

As summarized in Table 4, relatively direct evidence is mainly available for freeze–thaw–salt/chloride and freeze–thaw–sulfate exposure, whereas shrinkage–transport interaction is primarily supported by correlation-based evidence. Carbonation–chloride–freeze–thaw interaction in reinforced GPC remains largely a mechanism-based inference and therefore requires direct validation. On this basis, Figure 5 further synthesizes the evidence hierarchy, cross-mechanism interaction bridge and key validation needs for coupled deterioration in GPC/AAM systems.

### 5.1. Damage Amplification Under Coupled Freeze–Thaw and Chemical Attack

Among the multi-hazard pathways discussed in this review, freeze–thaw–salt/chemical attack has relatively direct experimental support, particularly for GPC/AAM systems exposed to freeze–thaw cycling combined with chloride, sulfate, seawater or other saline environments. Available studies indicate that the coupled deterioration is governed by the combined effects of salt type, exposure sequence, saturation degree, fiber bridging, interfacial stability, pore structure and matrix toughness [53,54,55,56,57,58]. Freeze–thaw cycling can generate microcracks and connected transport pathways that facilitate the ingress of chloride, sulfate and moisture, while subsequent salt crystallization, ion exchange, gel dealkalization/decalcification and pore coarsening can further aggravate cracking, scaling and mechanical degradation. Microstructural observations reported by Bao et al. [59] further support this feedback mechanism, showing that repeated low-pressure salt-attack and freeze–thaw exposure progressively increases crack density, fractured interfaces, pore defects and matrix loosening. These findings suggest that frost-induced cracking and saline ingress can interact reciprocally, leading to a system-dependent feedback process in which cracking accelerates aggressive-agent transport, while chemical and crystallization-related damage further promotes gel degradation and crack connectivity.

Studies on coal gangue–slag concrete, fly ash–slag GPC, geopolymer recycled concrete and high-toughness low-carbon GPC indicate that coupled freeze–thaw–salt deterioration is strongly dependent on exposure stage, saline environment and mixture composition [60,61,62,63,64]. Early exposure may show temporary mass stability or slight mass gain because of salt retention and secondary-product filling, whereas prolonged cycling progressively promotes surface scaling, crack interconnection, pore-structure deterioration and loss of matrix integrity. NaCl exposure generally intensifies frost-induced scaling, while binder composition, including slag incorporation, can modify the deterioration rate by altering matrix compactness, pore connectivity and crack resistance. These findings suggest that freeze–thaw–salt deterioration should be interpreted as a stage-dependent feedback process involving saline ingress, crystallization-related stress and progressive cracking, rather than by frost-heave pressure alone; importantly, early mass stability may conceal accumulating internal damage and should not be regarded as improved durability.

Accordingly, coupled damage models should avoid simplistic superposition of single-factor test results and instead incorporate interactive terms related to aggressive-agent transport, crack propagation, material composition, exposure sequence, interfacial toughness and initial shrinkage cracking. Future experimental designs should adopt sequential coupling schemes that replicate in-service deterioration pathways, including pre-shrinkage deformation, carbonation evolution, chloride penetration, sulfate attack and freeze–thaw cycling.

### 5.2. Promotion of Aggressive-Media Transport and Damage Propagation by Drying Shrinkage

Shrinkage–transport interaction is treated in this review as a Level II coupled pathway rather than as a fully verified coupled deterioration law for all GPC/AAM systems. Direct long-term tests that impose shrinkage history followed by aggressive exposure on concrete-scale GPC remain limited. Nevertheless, available studies on drying shrinkage, autogenous shrinkage, permeability and pore-structure evolution consistently indicate that shrinkage-induced microcracks can modify the initial transport boundary of GPC/AAM systems. External drying and internal self-desiccation generate restrained shrinkage stresses at material surfaces, interfacial transition zones and inherent defects; when local tensile stress exceeds the matrix tensile capacity, microcracks may initiate and connect with the pore network. As a result, the ingress of water, chloride, sulfate and CO_2_ may shift from pore-dominated diffusion to coupled crack–pore permeation, thereby increasing the risk of subsequent freeze–thaw, chemical and carbonation-related degradation.

Studies on drying shrinkage, restrained cracking and early-age deformation indicate that shrinkage behavior is jointly influenced by activator dosage, pore structure, internal water retention, autogenous shrinkage stress, elastic modulus development and curing environment [123,124,125,126]. Therefore, shrinkage evaluation should focus not only on deformation amplitude, but also on crack initiation, crack connectivity and the resulting change in transport boundaries.

Shrinkage-induced cracking can modify the intrinsic transport behavior of GPC/AAM matrices by changing pore connectivity, crack morphology and interfacial integrity. Correlation evidence from fiber-reinforced alkali-activated composites indicates that shrinkage and permeability are closely related, although fiber bridging may partially restrain crack propagation [127]. In addition, transport tests for alkali-activated materials are affected by pore-solution conductivity, ion binding capacity and applied electric fields, which makes conventional OPC-based charge permeability criteria unsuitable for direct application to GPC systems [128]. Therefore, the influence of shrinkage cracks on transport performance should be evaluated through integrated indicators, including crack width and connectivity, pore structure, interfacial condition and actual ion migration behavior. When paste- or mortar-scale shrinkage results are used to infer concrete-scale GPC durability, aggregate restraint, interfacial transition zones and size-dependent crack connectivity should also be explicitly considered.

Microscopically, drying and autogenous shrinkage are closely related to gel water status, pore-structure evolution and internal humidity variation. Studies on alkali-activated slag and related alkali-activated systems indicate that pore refinement, interlayer water migration, capillary pressure fluctuation, gel rearrangement, material age and ambient relative humidity jointly regulate shrinkage development [129,130,131]. These findings provide microstructural support for interpreting shrinkage as a transport-boundary modifying process rather than only as dimensional deformation.

At the paste scale, Chen et al. [122] showed that partial substitution of Na_2_SiO_3_ by NaAlO_2_ in alkali-activated slag reduced both drying and autogenous shrinkage and modified the associated pore structure. As illustrated in Figure 6, increasing NaAlO_2_ substitution reduced total porosity and shifted the pore-size distribution toward finer pores, accompanied by changes in reaction products such as hydrotalcite and tetranatrolite. These results support a composition-dependent relationship between activator chemistry, pore refinement and shrinkage mitigation. However, because the evidence was obtained from AAS paste, its extrapolation to concrete-scale GPC should account for aggregate restraint, ITZ characteristics and drying boundary conditions.

During long-term service, shrinkage, permeability and humidity boundary conditions should be considered together because they jointly affect moisture exchange and aggressive-agent transport. Seneviratne et al. [132] reported that the creep, shrinkage and permeation properties of geopolymer aggregate concrete vary with aggregate type, pore microstructure and curing regime, indicating that shrinkage damage assessment should not rely solely on early-age free shrinkage. Hossain and Sood [133] further showed that C-A-S-H/C-S-H binding gels and micro-restraint effects induced by PVA fibers can reduce shrinkage deformation and help preserve residual dynamic stiffness of high-calcium alkali-activated composites after freeze–thaw cycling. These studies suggest a correlation-supported link between shrinkage regulation, moisture transport and frost resistance, but this relationship still requires further validation in concrete-scale GPC under long-term coupled exposure.

From a transport-modeling perspective, Sirotti et al. [134] investigated the humidity-dependent moisture transport of NaOH-activated slag mortars using water-vapor desorption experiments and numerical modeling. As shown in Figure 7, the reliably modeled AAS systems exhibited a pronounced dependence of equivalent moisture diffusivity on relative humidity, with characteristic behavior differing from that of the Portland-cement references. However, model applicability was composition-dependent, and high-molarity mixtures were affected by deliquescence and associated uncertainties. These results suggest that moisture-transport parameters calibrated for OPC should not be transferred directly to GPC/AAM systems without material-specific validation and calibration.

In conclusion, drying and autogenous shrinkage may act as important pre-damage factors that increase the susceptibility of GPC to subsequent freeze–thaw, chemical attack and carbonation. By generating microcracks and modifying pore connectivity, shrinkage can alter the initial transport boundary of the matrix. Service-life models should therefore consider crack location, width and connectivity where such information is available, rather than relying solely on free-shrinkage values.

### 5.3. Reinforcement Corrosion Risk Associated with Carbonation, Chloride Ingress and Freeze–Thaw-Induced Cracking

The interaction among carbonation, chloride ingress and freeze–thaw-induced cracking in reinforced GPC is classified in this review as a Level III mechanism. This classification is adopted because most available studies focus on carbonation-induced corrosion, chloride-induced corrosion, steel passivation/depassivation or freeze–thaw damage separately, whereas direct experimental evidence on simultaneous or sequential carbonation–chloride–freeze–thaw coupling in reinforced GPC remains scarce. Therefore, the following discussion does not treat triple coupling as a fully verified deterioration law, but as a plausible service-risk pathway inferred from separate lines of evidence. Shrinkage- and frost-induced cracks may accelerate the ingress of CO_2_ and chloride ions. Carbonation lowers pore-solution pH and alters gel microstructure, whereas chloride elevates steel depassivation risk under reduced alkalinity and elevated moisture. Accordingly, durability evaluation for reinforced GPC cannot depend merely on carbonation depth or chloride diffusion coefficient; pore-solution chemistry, crack connectivity, moisture condition and steel electrochemical behavior need to be assessed collectively.

Available studies on reinforcement protection in GPC/AAM systems indicate that steel passivation cannot be explained by porosity or pH alone [65,66,67,68]. Pore-solution composition, sulfide content, soluble silicate–aluminate species, chloride threshold and steel–matrix interfacial features jointly affect passive-film stability and corrosion initiation. Therefore, carbonation depth and chloride concentration should be interpreted together with pore-solution chemistry and steel electrochemical response.

Upon chloride exposure, reinforcement corrosion in GPC/AAM systems is governed by the coupled effects of precursor chemistry, pore-solution buffering capacity, moisture state, chloride threshold and cover integrity rather than by any single electrochemical parameter [69,70,71,72]. Carbonation-induced alkalinity loss and changes in pore structure can further modify chloride transport and steel depassivation, while the relationships between corrosion current, polarization resistance and actual corroded area remain strongly material-dependent. Therefore, conventional pH- or chloride-threshold-based criteria developed for OPC cannot be directly transferred to GPC/AAM systems; a more reliable assessment should integrate pore-solution chemistry, transport resistance, cover cracking and direct electrochemical or corrosion measurements.

Certain GPC/AAM systems exhibit favorable steel-protection potential under chloride-rich conditions, yet such superiority is conditional upon pore-solution alkalinity, transport resistance and steel-matrix interfacial stability and cannot be over-generalized. Criado and Provis [73] reported that chloride-induced corrosion resistance of alkali-activated mortars is controlled by these three critical factors. Ichimiya et al. [74] proposed a performance-based approach combining chloride transport testing with direct quantification of rebar corrosion in fly ash–GGBS geopolymer mortars. As shown in Figure 8, their results showed weak correlations between rusted area and either compressive strength or chloride diffusion coefficient alone, indicating that transport resistance during the initiation stage cannot fully represent corrosion behavior during the propagation stage. Surface cracking observed after cyclic NaCl wetting and drying may additionally involve restrained shrinkage and therefore should not be attributed exclusively to corrosion-product expansion. These findings support a multi-indicator assessment integrating chloride transport, moisture history, crack development and direct reinforcement-corrosion measurements. Masi [75] also found that steel-protection efficiency of lightweight fly ash alkali-activated mortars is dominated by matrix resistivity, pore-solution chemistry and chloride migration characteristics.

In conclusion, carbonation–chloride–freeze–thaw deterioration in reinforced GPC should currently be regarded as a plausible but insufficiently validated multi-hazard pathway. Existing evidence supports a mechanistic sequence in which freeze–thaw- or shrinkage-related cracking facilitates CO_2_ and chloride ingress, carbonation reduces pore-solution alkalinity and alters gel chemistry, and chloride accumulation increases the risk of steel depassivation. However, transport indicators alone cannot fully represent corrosion propagation, while observed cracking may arise from multiple sources rather than reinforcement corrosion alone. Therefore, direct sequential tests integrating pre-damage, carbonation, chloride exposure and steel electrochemical monitoring are required to distinguish the contribution of each process and to establish a reliable link between transport deterioration, depassivation, and corrosion propagation.

### 5.4. Multi-Indicator Evaluation and Service-Life Prediction Framework

Under multi-hazard conditions, GPC durability evaluation should evolve from simple comparison of individual performance indices toward systematic service-life prediction. Mass loss ratio, strength retention, relative dynamic elastic modulus, chloride diffusion coefficient, water absorption and drying shrinkage, together with carbonation depth and steel electrochemical parameters, characterize distinct damage stages and mechanisms. These indicators function heterogeneously rather than equivalently and should be integrated into a progressive analytical framework covering volume deformation and cracking, enhanced mass transport, gel degradation, interfacial defect evolution, steel depassivation and macroscopic performance degradation.

From an engineering design perspective, the practical application of GPC should not rely only on material-level durability indicators, but should also satisfy mechanical equivalence and member-scale load-bearing requirements comparable to those of OPC concrete under the same structural function and exposure condition [135,136,137]. For mixture proportioning, the minimum reported parameters should include precursor type and calcium level, activator characteristics, liquid-to-binder ratio, aggregate gradation, curing regime, workability, density, compressive strength, elastic modulus and tensile or flexural strength. For structural element design, member geometry, reinforcement ratio, cover thickness, loading mode, bond behavior, confinement condition and axial, flexural or shear capacity should be verified together. Therefore, GPC structural application should follow a performance-based route involving trial mixture optimization, mechanical equivalence verification, durability equivalence assessment and member-scale load-bearing validation.

From a degradation-mitigation perspective, appropriate protection methods should also be considered to reduce aggressive-agent ingress and delay the primary deterioration processes of GPC/AAM systems [138,139]. Potential strategies include siliceous or silane-based coatings, hydrophobic treatment, crack-control measures, internal curing, air-void optimization and fiber/nanomaterial reinforcement. Among them, silicate- or silane-based reagents are relevant because hydrolysis and condensation reactions can form silica-rich or Si–O–Si networks, which may reduce capillary pore connectivity and limit the ingress of water, chloride ions, sulfate ions and CO_2_. However, their long-term effectiveness in GPC/AAM systems should be further verified under wet–dry cycling, freeze–thaw exposure, sulfate/acid attack, carbonation and reinforcement corrosion conditions.

Specifically, mass loss rate primarily quantifies surface scaling and applies to the late-stage evaluation of acid attack, salt freeze–thaw damage and sulfate attack. Strength retention reflects the degradation of bearing capacity yet exhibits low sensitivity to early transport variations. Relative dynamic elastic modulus enables the early detection of internal cracking and structural integrity decay. Chloride diffusion coefficient, water absorption, gas diffusion coefficient and passed charge characterize the matrix transport permeability to aggressive substances, while the influences of pore-solution conductivity, ion binding behavior and crack connectivity must be adequately considered. Drying shrinkage, autogenous shrinkage and crack width serve as critical initial damage indicators, whereas pore-solution pH, gel microstructure, elemental leaching and steel electrochemical status support the assessment of carbonation deterioration and reinforcement protection performance.

Chidiac et al. [76] established a probabilistic service-life prediction model for chloride-induced corrosion of alkali-activated concrete, indicating that uncertainties in diffusion coefficient, cover thickness, surface chloride concentration and critical chloride threshold can substantially affect corrosion initiation time. Amleh et al. [77] constructed a probabilistic corrosion initiation model for fly ash–slag blended GPC bridge decks and adopted Monte Carlo analysis to clarify the influences of temperature, relative humidity, cover thickness and chloride diffusivity on corrosion probability. These studies suggest that GPC/AAM durability evaluation should advance from qualitative phenomenological interpretation to quantitative parameter extraction, model input definition and service-life calibration.

Available service-life prediction studies for GPC/AAM systems remain limited and mainly focus on chloride-induced corrosion initiation. Their application scope, model type, main input parameters and validation limitations are summarized in Table 5.

As summarized in Table 5, existing service-life models provide useful probabilistic frameworks for chloride-induced corrosion initiation, but they still do not fully incorporate exposure sequence, shrinkage- or freeze–thaw-induced cracking, carbonation state, chloride binding/release and steel electrochemical evolution. Future models should therefore move from single-transport-parameter prediction toward multi-indicator, chemistry-specific and field-calibrated frameworks for coupled service environments.

In conclusion, multi-hazard durability assessment of GPC requires a hierarchical framework integrating indicator classification, parameter quantification, model implementation and service-life prediction. Mass loss and relative dynamic elastic modulus can be used to characterize freeze–thaw deterioration and surface scaling; chloride diffusion coefficient, water absorption and gas permeability can reflect aggressive-agent transport capacity; shrinkage strain and crack width can describe initial pre-damage levels; and carbonation depth, pore-solution pH and steel electrochemical responses can be used to evaluate reinforcement protection performance. Future research should establish chemistry-specific parameter databases for low-calcium GPC systems and high-calcium or slag-rich AAM systems, and calibrate accelerated laboratory results using long-term field exposure data.

## 6. Conclusions and Perspectives

(1) The durability of GPC cannot be generalized as inherently superior to that of OPC. Its performance depends strongly on precursor chemistry, calcium content, activator formulation, curing history and exposure conditions. Low-calcium fly ash- or metakaolin-based GPC and high-calcium or slag-rich AAM systems should therefore be evaluated separately. Across different exposures, pore connectivity, crack development, gel stability and pore-solution chemistry are more informative than any single macroscopic indicator.

(2) Freeze–thaw and chemical attack show relatively well-established deterioration pathways. Freeze–thaw damage develops through pore-water phase change, internal cracking and progressive transport-pathway formation, whereas chemical attack is governed mainly by ion ingress, dealkalization, dealumination/decalcification, elemental leaching and pore coarsening. Mass loss or strength retention alone cannot fully describe these processes. Shrinkage and carbonation are also important because they alter the initial crack network, transport conditions and reinforcement-protection environment before or during subsequent exposure.

(3) Evidence for multi-hazard deterioration is not equally strong. Direct experimental support is currently strongest for freeze–thaw combined with chloride/salt or sulfate exposure. Shrinkage–transport interaction is mainly supported by correlations between cracking and permeability, while the proposed pathway linking carbonation, chloride ingress and freeze–thaw cracking to reinforcement corrosion remains largely a mechanism-based inference. These latter pathways should therefore be regarded as plausible service risks rather than established coupled deterioration laws.

(4) The main unresolved issue is the translation of accelerated laboratory results to long-term field performance. Current testing protocols, exposure sequences and service-life models remain insufficiently calibrated against natural exposure. Future work should therefore prioritize three tasks: standardized sequential multi-hazard testing, field calibration of transport and corrosion models, and chemistry-specific durability databases for different GPC/AAM systems. These steps are necessary before laboratory-derived deterioration mechanisms can be used reliably for service-life prediction and engineering design.

## Figures and Tables

**Figure 1:**
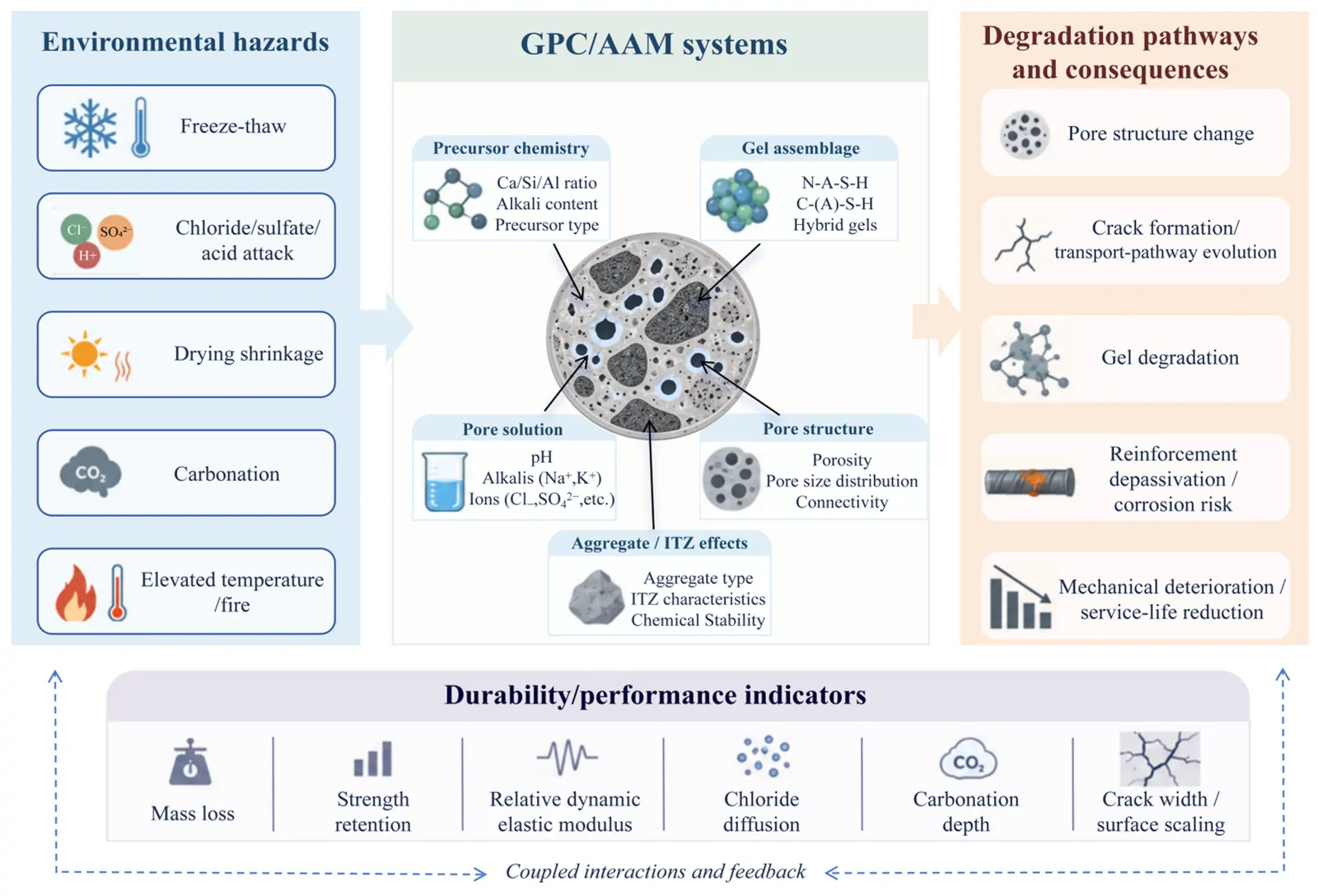
Multi-hazard deterioration framework of GPC/AAM systems. AI-assisted preparation: ChatGPT (OpenAI) was used only to assist with the initial conceptual and visual framework of this schematic figure. The final figure was manually redrawn, edited, and finalized by the authors using Microsoft PowerPoint. All scientific content and labels were determined and verified by the authors.

**Figure 2:**
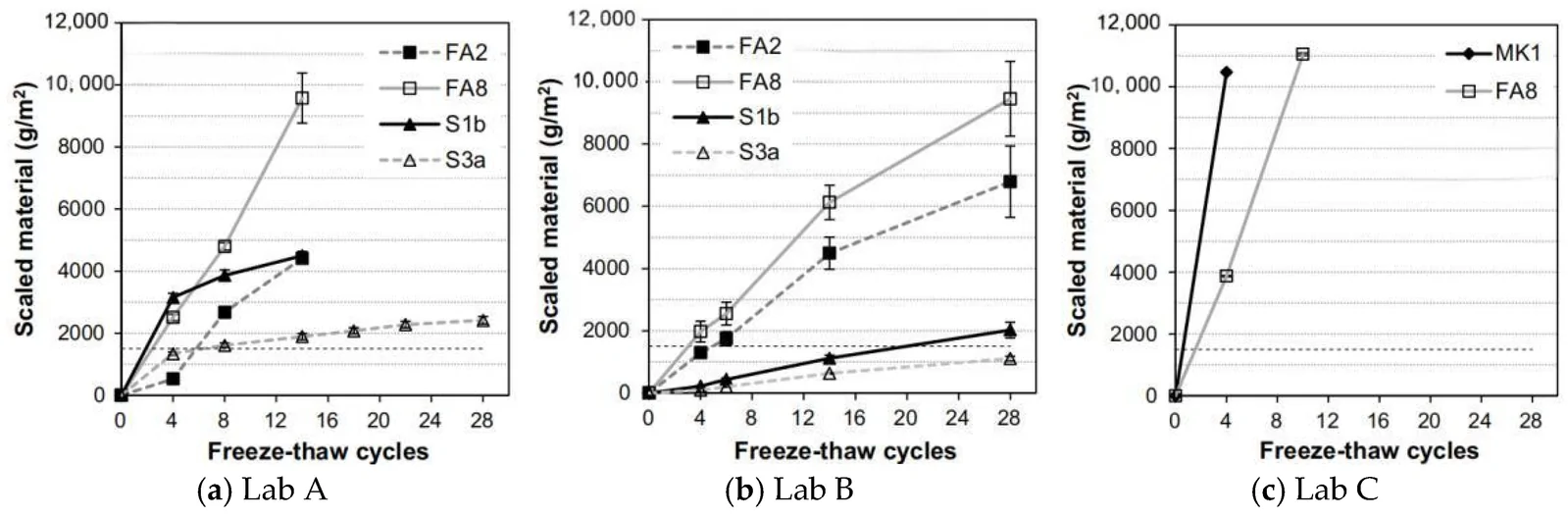
Cumulative scaled material during capillary suction–deicing salt freeze–thaw (CDF) testing of alkali-activated concretes after 28 d curing in three participating laboratories: (**a**) Laboratory A, (**b**) Laboratory B, and (**c**) Laboratory C. FA2 and FA8 denote high- and moderate-performance fly ash-based concretes, respectively; S3a and S1b denote high- and moderate-performance slag-based concretes, respectively; MK1 denotes metakaolin-based concrete.The horizontal dashed line indicates the commonly used CDF acceptance criterion of 1500 g/m^2^ after 28 freeze–thaw cycles. Adapted from Ref. [31], using panels (a), (c), and (d) of the source figure, under the terms of the CC BY 4.0 license.

**Figure 3:**
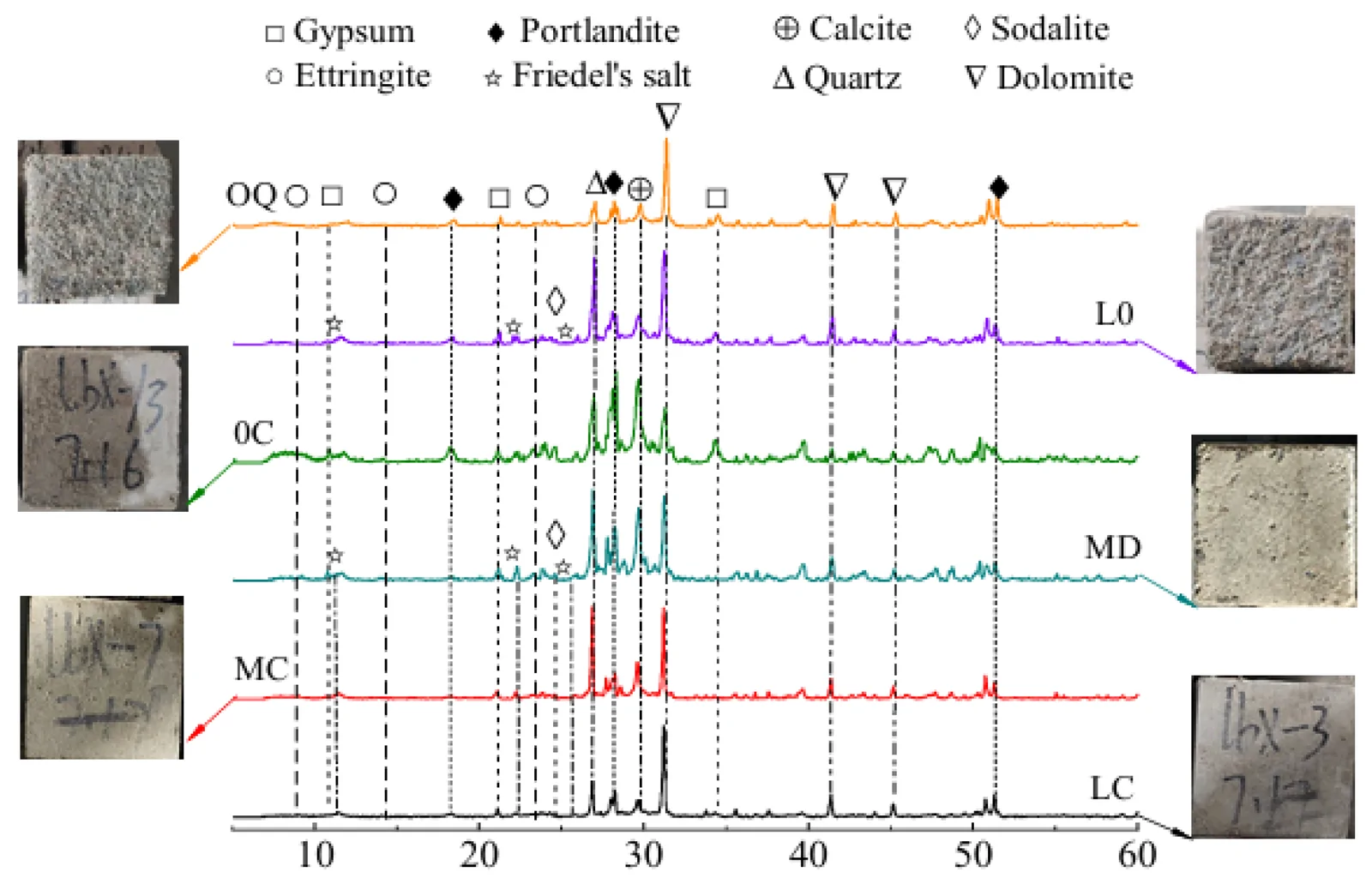
XRD patterns and corresponding specimen appearance of slag-rich alkali-activated concrete after 90 d of sulfate immersion. LC, MC and MD denote mixtures containing 2/4/4% NaCl and 7.5/7.5/10% gypsum, respectively; 0C contains gypsum without NaCl; L0 contains NaCl without gypsum; and OQ contains neither NaCl nor gypsum. Slag content was fixed at 75%, with quicklime accounting for the remaining activator fraction. The identified crystalline phases include portlandite, Friedel’s salt, calcite, quartz, gypsum, ettringite, sodalite and dolomite. Adapted from Ref. [115] under the terms of the CC BY 4.0 license.

**Figure 4:**
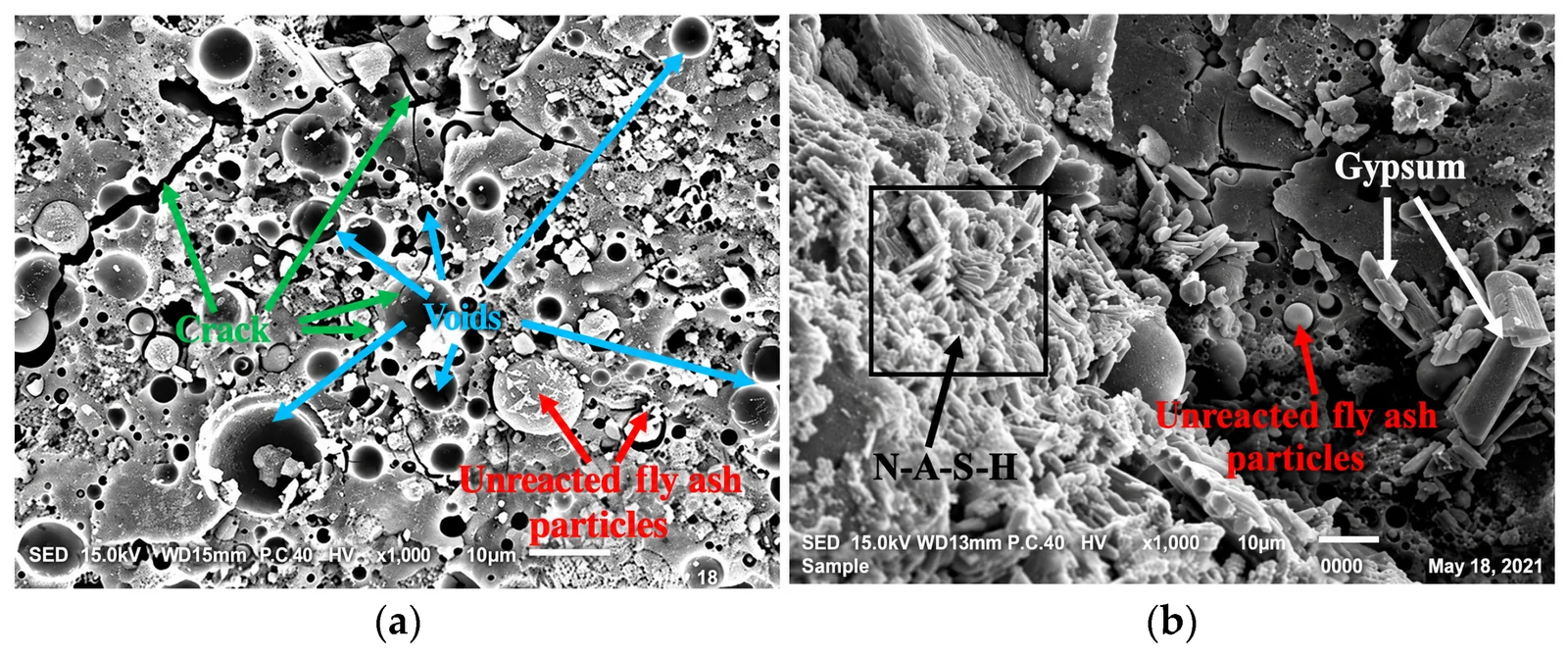
SEM images of low-calcium Class F fly ash-based GPC (F-8) before and after sulfuric-acid exposure: (**a**) unexposed specimen and (**b**) specimen after 98 d of cyclic exposure consisting of 6 d immersion in sulfuric acid at pH = 1 followed by 1 d drying. F-8 denotes the Class F fly ash-based mixture activated with 8 M NaOH, with Na2SiO3/NaOH = 1.5. Adapted from panels (b) and (c) of the original figure in Ref. [116], under the terms of the CC BY 4.0 license.

**Figure 5:**
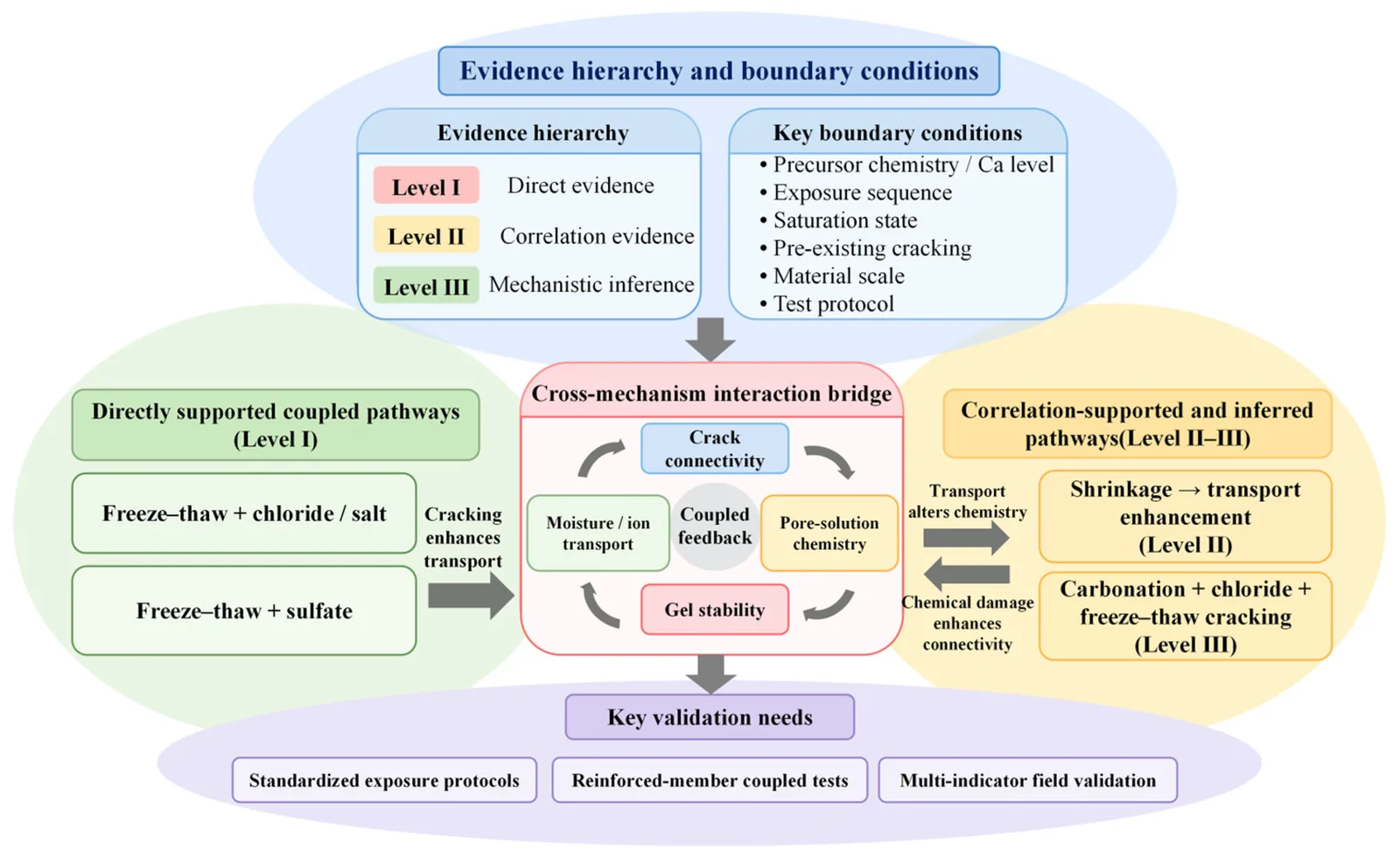
Evidence hierarchy and coupled deterioration pathways in GPC/AAM systems. ChatGPT (OpenAI) was used only to assist with the initial conceptual and visual framework of this schematic figure. The final figure was manually redrawn, edited, and finalized by the authors using Microsoft PowerPoint. All scientific content and labels were determined and verified by the authors.

**Figure 6:**
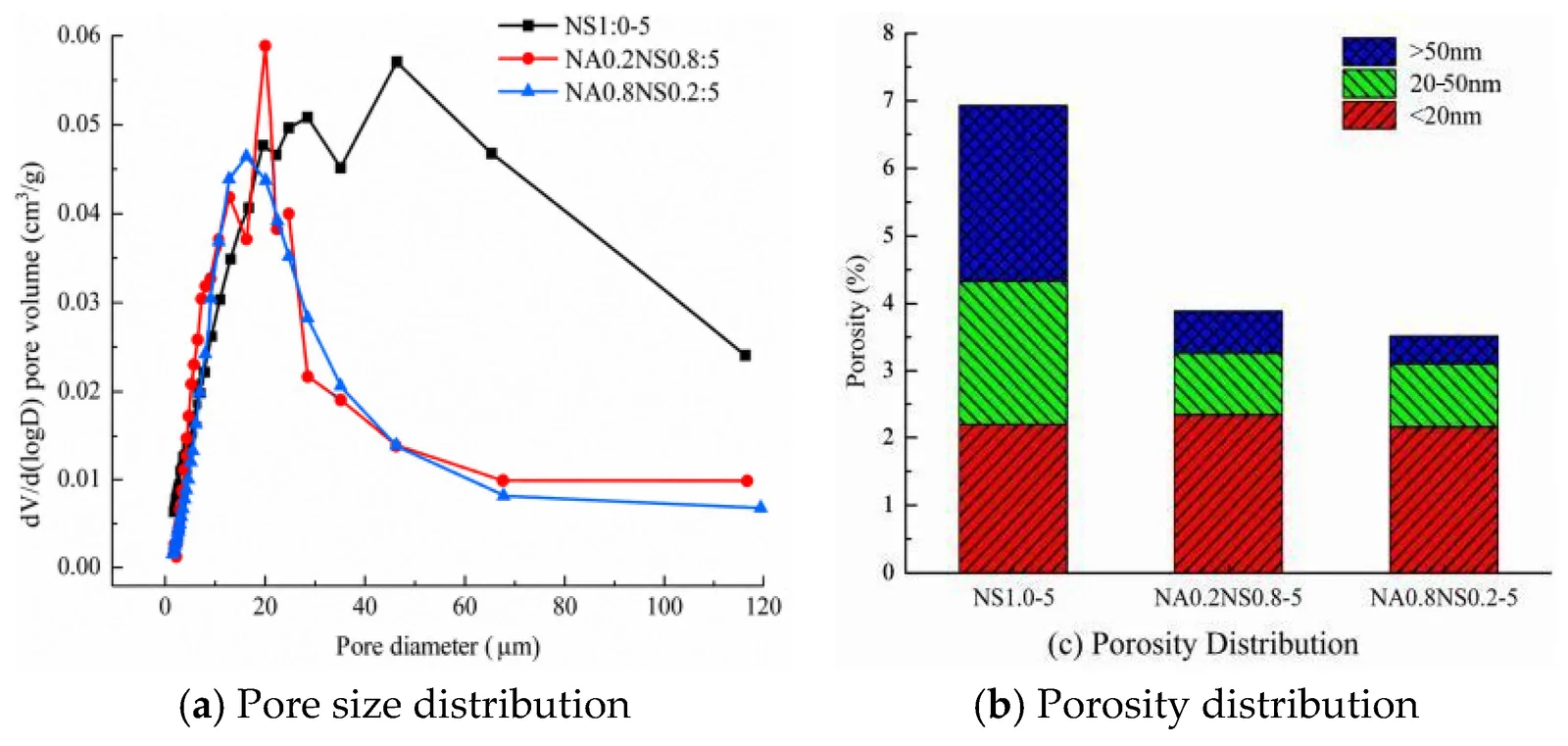
Effect of NaAlO_2_ substitution on the pore structure of alkali-activated slag paste: (**a**) pore-size distribution and (**b**) porosity partition. NS1.0-5 denotes 0% NaAlO_2_ and 100% Na_2_SiO_3_ at 5% Na_2_O; NA0.2NS0.8-5 denotes 20% NaAlO_2_ and 80% Na_2_SiO_3_ at 5% Na_2_O; and NA0.8NS0.2-5 denotes 80% NaAlO_2_ and 20% Na_2_SiO_3_ at 5% Na_2_O. Adapted from the pore-size distribution and porosity plots reported in Ref. [122], under the terms of the CC BY 4.0 license.

**Figure 7:**
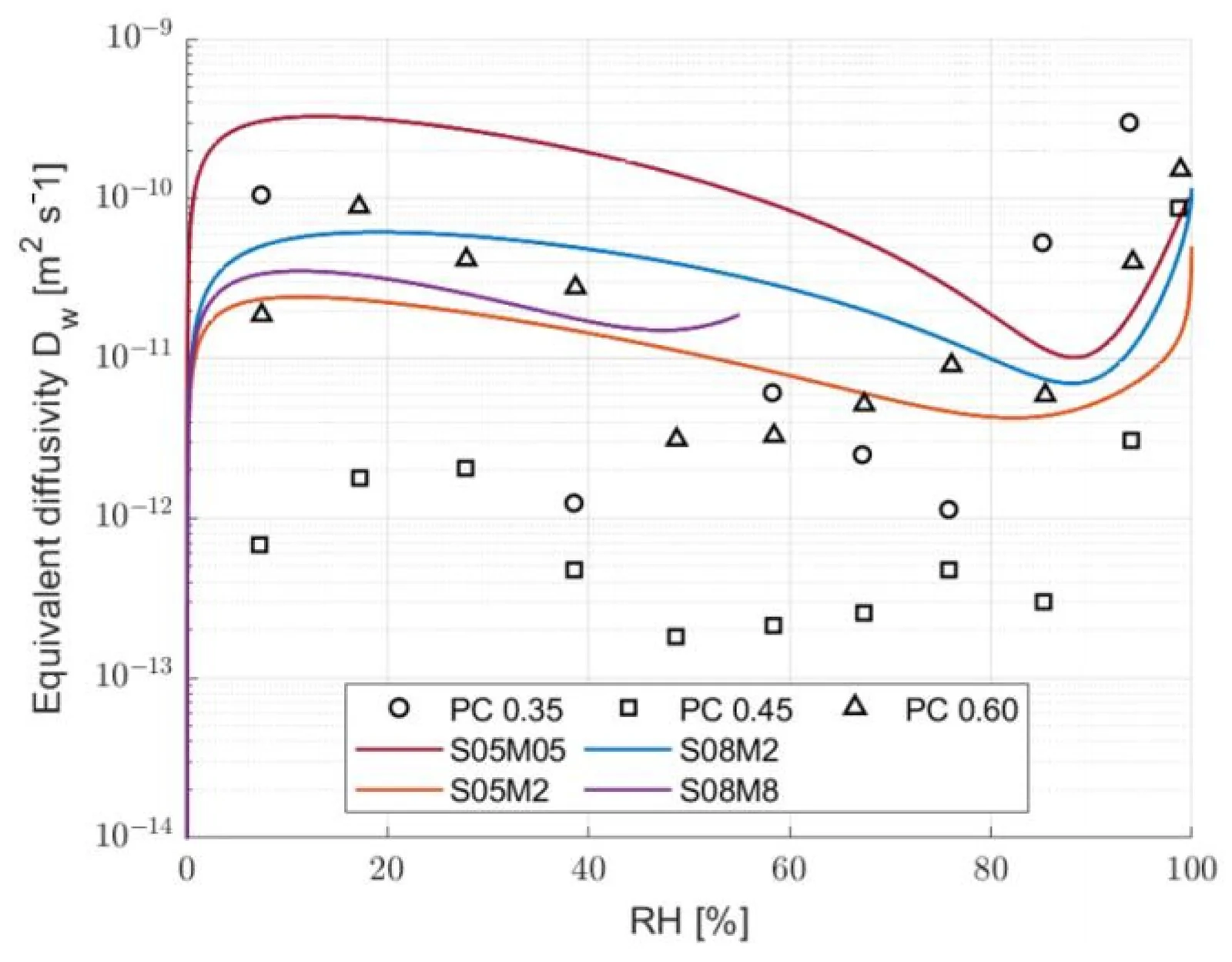
Equivalent moisture diffusivity as a function of relative humidity for selected NaOH-activated slag mortar compositions and Portland-cement reference systems. S05 and S08 denote solution-to-binder ratios of 0.5 and 0.8, respectively, while M05, M2 and M8 denote NaOH concentrations of 0.5, 2 and 8 M, respectively. PC 0.35, PC 0.45 and PC 0.60 denote Portland-cement reference systems with water-to-binder ratios of 0.35, 0.45 and 0.60, respectively. RH denotes relative humidity. Adapted from Ref. [134] under the terms of the CC BY 4.0 license.

**Figure 8:**
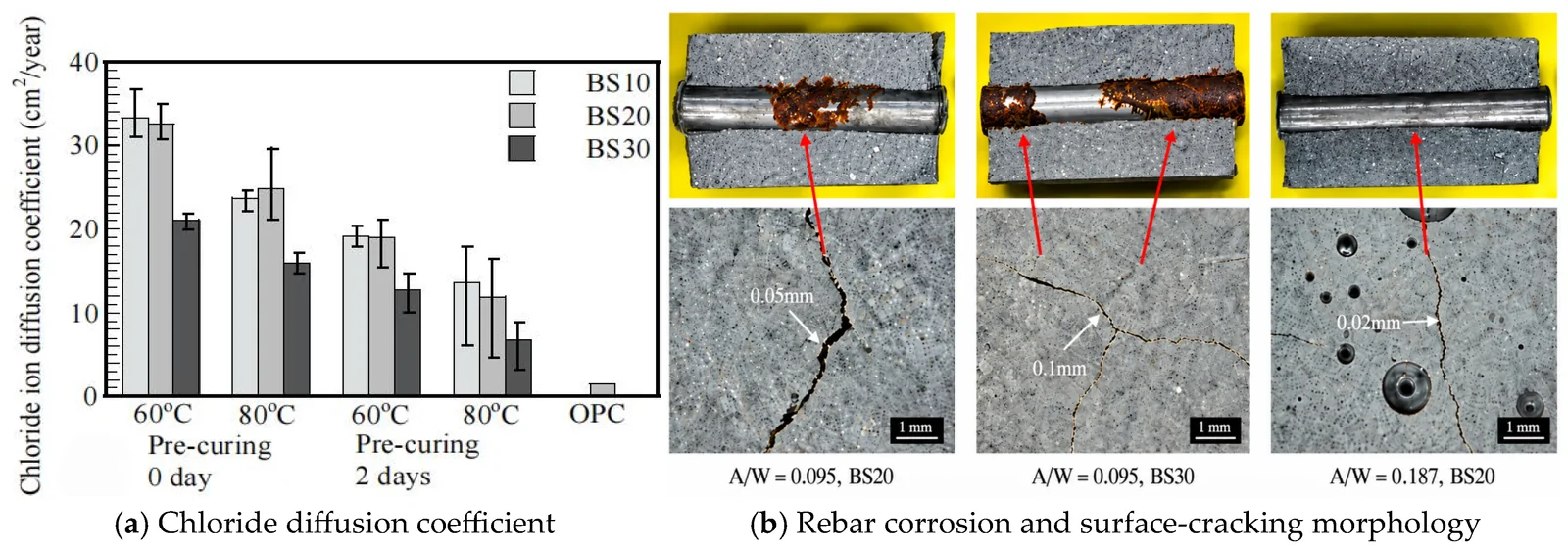
Chloride transport and reinforcement response of fly ash–GGBS geopolymer mortars: (**a**) chloride ion diffusion coefficients under different curing conditions for A/W = 0.126; (**b**) representative rebar corrosion and surface-cracking morphologies of selected mixtures. BS10, BS20 and BS30 denote GGBS replacement ratios of 10%, 20% and 30% by volume of the total precursor powder, respectively. A/W denotes the alkali-component-to-water ratio in the activating solution. OPC denotes the Portland-cement reference. Adapted from Ref. [74] under the CC BY 4.0 license.

**Table 1:** Comparison of representative reviews and the contribution of this review.

Ref.	Year	Material System	Main Focus	Coupled Deterioration/Corrosion/Service-Life Coverage	Remaining Gap and Contribution of This Review
Chen et al. [4]	2021	GPC and OPC	Durability under aggressive environments	Multiple environments discussed; limited service-life focus	This review further links cracking, transport, gel degradation and reinforcement risk into a multi-hazard damage chain.
Zhang [5]	2024	Low-carbon GPC	General durability assessment	Multi-hazard and multi-indicator concepts discussed	This review further distinguishes direct coupled evidence from mechanism-based inference.
Wong [6]	2022	GPC	Acid, salt, carbonation and freeze–thaw durability	Exposure conditions mainly discussed separately	This review emphasizes system-dependent mechanisms in Low-calcium GPC and high-calcium or slag-rich AAM systems.
Tian et al. [7]	2021	Geopolymer binders and GPC	General durability of geopolymers	Broad durability coverage; limited coupling classification	This review focuses on coupled deterioration under realistic multi-hazard service environments.
Luhar et al. [8]	2022	Fly ash-based geopolymers	Material properties and performance	Coupled durability not central	This review extends the discussion to low-calcium GPC and high-calcium or blended AAM systems.
Provis [9]	2018	AAMs	Fundamental chemistry of alkali activation	Durability coupling not central	This review uses AAM chemistry to interpret durability degradation in concrete-scale GPC.
Zhang et al. [10]	2025	Geopolymer gel composites	Freeze–thaw resistance and models	Mainly freeze–thaw-related	This review places freeze–thaw damage within a broader framework including chemical attack, shrinkage, carbonation and corrosion.
Aiken et al. [11]	2022	Alkali-activated binders	Acid resistance and testing methods	Mainly acid exposure	This review compares acid, sulfate and chloride attack and links chemical degradation with transport and freeze–thaw damage.
Zhao et al. [12]	2024	GPC	Carbonation behavior and modeling	Carbonation modeling emphasized	This review links carbonation with pore-solution alkalinity, chloride ingress, steel depassivation and service-life assessment.
Xu et al. [13]	2024	Reinforced AAMs	Steel passivation and depassivation	Reinforcement corrosion emphasized	This review incorporates reinforcement corrosion into a multi-hazard deterioration framework and identifies evidence gaps.

**Table 2:** Representative material systems, conditioning variables, exposure conditions and durability responses.

Ref.	Material System	Ca Level	Binder/Activator Feature	Curing/Exposure Condition	Main Durability Response
[18,20]	Fly ash-based GPC; GPC–OPC comparison	Low-Ca	Aluminosilicate gel-dominated	Standard curing; acid, sulfate or chloride exposure	Durability is governed by gel chemistry, pore structure and transport resistance.
[22,23,24]	AAS and slag-rich AAM mortars	High	C-A-S-H/C-(N)-A-S-H related gels	Freeze–thaw exposure	Frost resistance depends on pore refinement, gel stability and strength retention.
[25,26,27]	Fly ash/slag-based geopolymers and GPC	Low-Ca	Precursor proportion and mineral admixtures varied	Freeze–thaw cycles	Mass loss, RDEM and residual strength reflect staged frost deterioration.
[24,28,29,30,31]	Alkali-activated concrete and mortars	High-Ca	Mixture design and air-void system modified	Freeze–thaw or CDF scaling tests	Air-void stability and pore connectivity control frost durability.
[32,33,34,35,36]	Fly ash/slag GPC, AAS concrete and paste systems	High-Ca	Activator dosage and slag fraction varied	Drying or autogenous shrinkage	Shrinkage is associated with self-desiccation, capillary stress and restrained cracking.
[11,37,38,39,40,41,42,43,44,45,46,47]	GPC, geopolymer mortar and AAM concrete	System-dependent	Fly ash, slag, metakaolin or blended precursors	Acid, sulfate or chloride attack	Dealkalization, leaching, secondary products and pore coarsening dominate damage.
[12,13,48,49,50,51,52]	GPC and AAM carbonation systems	System-dependent	N-A-S-H, C-A-S-H or C-(N)-A-S-H related gels	Natural or accelerated carbonation	Carbonation changes alkalinity, gel assemblages and steel-protection capacity.
[53,54,55,56,57,58,59,60,61,62,63,64]	GPC/AAM under coupled environments	System-dependent	Fiber, slag, recycled aggregate or low-carbon mixtures	Freeze–thaw with salt, chloride, sulfate or seawater	Coupled damage depends on salt type, exposure sequence, saturation and matrix toughness.
[65,66,67,68,69,70,71,72,73,74,75]	Reinforced GPC/AAM systems	System-dependent	Steel–matrix interface considered	Carbonation, chloride or corrosion exposure	Steel depassivation is affected by alkalinity, chloride ingress and electrochemical response.
[76,77]	Alkali-activated concrete and GPC bridge decks	High-Ca	Transport parameters modeled	Chloride-induced corrosion modeling	Service life depends on diffusivity, cover depth, chloride threshold and climate factors.

**Table 3:** Mechanisms, recommended indicators, and critical boundary conditions for major durability hazards.

Hazard	Dominant Mechanism	Recommended Indicators	Critical Boundary Conditions
Freeze–thaw cycling	Pore-water freezing, hydraulic pressure, pore coarsening and ITZ cracking	Mass loss, RDEM, UPV, residual strength, pore size, air-void parameters	Saturation degree, freezing regime, specimen size, pore connectivity
Chloride attack	Chloride ingress, ion binding/release, pore-solution change and steel depassivation risk	Chloride diffusion coefficient, chloride profile, passed charge, steel potential/current	Pore-solution conductivity, chloride binding, cover cracking, alkali content
Sulfate attack	Sulfate ingress, secondary products, crystallization stress and gel degradation	Mass change, strength retention, sulfate depth, XRD/SEM, pore structure	Sulfate type, wet–dry cycles, calcium level, exposure duration
Acid attack	Ion exchange, dealkalization, dealumination/decalcification and elemental leaching	Mass loss, strength loss, pH variation, leached ions, SEM/XRD/FTIR	Acid type, concentration, Ca content, gel stability
Drying/autogenous shrinkage	Self-desiccation, capillary pressure, gel water loss and restrained cracking	Shrinkage strain, crack width, internal RH, permeability, elastic modulus	Activator dosage, slag content, curing RH, aggregate restraint
Carbonation	CO_2_ ingress, alkalinity loss, alkali leaching, gel decalcification and carbonate precipitation	Carbonation depth, pore-solution pH, soluble alkalis, phase assemblage, steel electrochemistry	CO_2_ concentration, RH, Ca level, phenolphthalein reliability
Elevated temperature/fire	Moisture migration, pore-pressure build-up, gel dehydration, phase transformation, thermal cracking and spalling	Mass loss, residual strength/modulus, crack and spalling characteristics, pore structure, XRD/TGA, SEM	Heating rate, peak temperature, exposure duration, cooling regime, initial moisture, aggregate type and precursor/Ca chemistry

**Table 4:** Evidence levels and interaction characteristics of coupled exposure pathways.

Coupled Pathway	Evidence Level	Representative Refs.	Mechanistic Interpretation	Key Boundary Conditions	Current Limitation
Freeze–thaw + chloride/salt	Level I	[53,54,55,56,57,58,59,60,61,62,63,64,120,121]	Freeze–thaw cracking and pore coarsening facilitate saline ingress, while salt transport, crystallization and chemical alteration further promote scaling and crack connectivity.	Salt type/concentration; saturation state; exposure sequence; binder chemistry; curing and freeze–thaw protocol	Direct coupled evidence is available, but results remain strongly system- and protocol-dependent.
Freeze–thaw + sulfate	Level I	[54,55]	Frost-induced cracking increases sulfate accessibility, whereas sulfate-related reaction, crystallization and gel degradation can further weaken the matrix and promote crack propagation.	Sulfate species/concentration; saturation state; exposure sequence; Ca level; pore structure	Long-term concrete-scale and reinforced-member evidence remains limited.
Shrinkage → transport enhancement	Level II	[122,123,124,125,126,127,128,129,130,131,132,133,134]	Shrinkage-induced microcracks and increased pore/crack connectivity can enhance moisture, ion and CO_2_ transport, thereby modifying subsequent deterioration susceptibility.	Shrinkage type; RH; aggregate restraint; curing history; crack width/connectivity; material scale	The interaction is mainly correlation-supported; direct sequential multi-hazard validation remains scarce.
Carbonation + chloride + freeze–thaw-induced cracking	Level III	[13,65,66,67,68,69,70,71,72,73,74,75]	Freeze–thaw-induced cracking may accelerate CO_2_ and Cl^−^ ingress; carbonation reduces alkalinity and alters binding conditions, while chloride accumulation may increase reinforcement depassivation and corrosion risk.	Pre-existing cracking; RH/saturation; carbonation history; chloride exposure; cover depth; precursor chemistry	The pathway is primarily mechanism-based inference; direct triple-coupled tests on reinforced GPC are largely unavailable.

**Table 5:** Service-life prediction models and validation status.

Ref.	System	Degradation Process	Model Type	Main Inputs	Validation/Limitation
[76]	Alkali-activated concrete	Chloride-induced corrosion initiation	Probabilistic service-life model	Diffusion coefficient, cover depth, surface chloride, chloride threshold	Provides a probabilistic framework; field calibration for different AAM systems remains limited.
[77]	Fly ash–slag GPC bridge decks	Climate-dependent chloride corrosion initiation	Monte Carlo probabilistic model	Temperature, RH, cover depth, chloride diffusivity	Considers climate uncertainty; coupled cracking, carbonation and freeze–thaw effects need validation.
Future framework proposed	GPC/AAM under multi-hazard exposure	Conceptual multi-indicator service-life framework	Multi-indicator service-life framework	Damage indices, transport parameters, crack state, carbonation state, pore solution, steel electrochemistry	Requires standardized sequential coupling tests and long-term field calibration.

## Data Availability

No new data were created or analyzed in this study. Data sharing is not applicable to this article.
